# Effects of Inbreeding on Genetic Characteristic, Growth, Survival Rates, and Immune Responses of a New Inbred Line of *Exopalaemon carinicauda*

**DOI:** 10.1155/2020/5735968

**Published:** 2020-01-03

**Authors:** Jiajia Wang, Jitao Li, Qianqian Ge, Zhao Chen, Jian Li

**Affiliations:** ^1^Key Laboratory for Sustainable Utilization of Marine Fisheries Resources, Ministry of Agriculture and Rural, Yellow Sea Fisheries Research Institute, Chinese Academy of Fishery Sciences, Qingdao, China; ^2^Function Laboratory for Marine Fisheries Science and Food Production Processes, Qingdao National Laboratory for Marine Science and Technology, Qingdao, China

## Abstract

The *Exopalaemon carinicauda* could be a useful crustacean laboratory animal in many research fields. We newly established an inbred line of *Exopalaemon carinicauda* named EC4 inbred line by brother×sister mating and keeping to *F*_11_ generation. Trends in heterozygosity in the process of producing EC4 inbred line were examined through the characterization of polymorphisms based on gene frequencies of SNP and EST-SSR loci. The results demonstrated that the number of alleles (*N*), observed heterozygosity (*Ho*), expected heterozygosity (*He*), and polymorphism information content (PIC) gradually decreased with the increase of inbreeding generations. The genetic detection results indicated that 9 (29.03%, 9/31) of the SNP loci and 15 (32.61%, 15/46) of the EST-SSR loci were homozygous in *F*_11_ generation of EC4 inbred line. The variation of the growth-related traits, the immune responses, and antioxidant status were described in experimental full-sibling inbred populations of *E. carinicauda* at five levels of inbreeding coefficient (*F* = 0.785, *F* = 0.816, *F* = 0.859, *F* = 0.886, *F* = 0.908) under controlled laboratory conditions. The body weight, body length, and survival rate in EC4 inbred line of all generations were less than the control population. Inbreeding affected the antibacterial activity, phenoloxidase (PO) activity, and superoxide dismutase (SOD) which decreased at the eleventh generation of EC4 inbred line. This study demonstrated that inbreeding had a negative effect on the economic traits and immune response, but our inbred line was established successfully until *F*_11_ and confirmed by genetic detection using SNP and EST-SSR loci.

## 1. Introduction

The laboratory animal is the basis and vital condition for life science research. Inbred strains of laboratory animal are frequently used to improve the reproducibility, stability, and reliability of animal-based experiments, which also provide standardized animal models to have reproducible experimental results using the same material from different researchers around the world [1]. Various inbred strains of laboratory animals have been used for promoting the development of genetics [2], oncology [3], and immunology [4]. Mammals, especially mice and rats, have severed for the most important laboratory animal; nevertheless, animals of other species are also expected to provide valuable information that cannot be obtained in these animals. Arthropod species account for over 80 percent of all known living animal species, and the crustacean which belongs to arthropod comprises almost 67,000 described species [5]; therefore, the cultivation of crustacean inbred laboratory animals is of great significance for the scientific research of aquatic crustacean.

The ridgetail white prawn *Exopalaemon carinicauda*, belonging to the Palaemonidae family of crustacea, is one of the major commercial mariculture species naturally distributed in China [6], which contributes one-third of the gross outcome of the polyculture ponds in eastern China [7]. Meanwhile, the *E. carinicauda* has a high nutritional value with high in protein, low in fat, and rich minerals. *E. carinicauda* has some characteristics to be a potential ideal laboratory animal for research on the genetics, pharmacology, toxicology, and disease research of aquatic crustacean, such as transparent body, moderate size, big egg (egg diameter ranges from 0.57 mm to 1.08 mm), short reproductive cycle, and good environmental adaptability [8–11]. Furthermore, *E. carinicauda* has a short reproductive cycle of only 2-3 months all years round and has a good environmental adaptability, which can survive at the temperature of 2-38°C, the salinity of 4-35, and the pH of 5.24-10.01 [12], and Liang et al. provide an artificial breeding technology in the laboratory [13]. Therefore, *E. carinicauda* would be an ideal species to be a crustacean inbred laboratory animal.

Inbred mating within a closed, small population tends to accelerate the loss of gene diversity and reduce the heterozygosity of genes in a population, ultimately leads to complete homozygosity observed from molecular markers [14]. Inbreeding also results in a decline in the mean phenotypic values of some traits, mainly including those related to fitness and physiological efficiency in normal outbreeding species, which is known as inbreeding depression [15, 16]. Not all species showed inbreeding depression for all characters, but almost all showed inbreeding depression for most characters [17]. Inbreeding depression has been reported for many different traits, including growth [18], survival [19], stress resistance [20], and reproductive traits [21, 22]. The magnitude of inbreeding depression on the fundamental economic traits may vary from species to species, also including aquaculture species. Moss et al. found no effect of inbreeding on growth trait in *P. vannamei* [23], while Luo et al. estimated a significant effect of inbreeding on growth in *Fenneropenaeus chinensis* [24]. Keys et al. estimated negative regression coefficients of growth and survival on inbreeding level, but not statistically significant in *P. japonicus* [25]. Ren et al. found that a high level of inbreeding could severely reduce the immune responses and antioxidant status of *P. trituberculatus* [20], but Luo et al. found no significant effect of inbreeding on survival time after challenge with white spot syndrome virus (WSSV) [24]. Therefore, it is necessary to estimate the likely effects of inbreeding on the fundamental economic traits and physiological health in *E. carinicauda* to ensure the successful establishment of inbred line.

Since 2009, our laboratory has begun to research on the breeding of inbred lines in *E. carinicauda*, for breeding crustacean laboratory animal. At present, the way of full-sibling mating has been handed down to the eleventh generation. This study examined the genetic characteristics of a new inbred line (EC4 inbred line) by molecular genetic method and investigated the effects of different levels of inbreeding on growth traits, survival, and morphological character for inclusion in the foundation of laboratory animal experiment in *E. carinicauda*.

## 2. Materials and Methods

### 2.1. Establishment of Inbred Lines and Control Population of *E. carinicauda*

Since 2009, our laboratory has begun to research on the breeding of inbred line of *E. carinicauda*, for breeding crustacean laboratory animal at the Yellow Sea Fisheries Research Institute using two wild, geographically distinct, Chinese populations (Jiaozhou Gulf and Laizhou Gulf). In both mice and medaka, over 10 inbred strains have been maintained by full-sib-pair mating for more than 20 generations and the use of these animals as vertebrate model systems [26, 27]. Comparable crustacean inbred strains have not been available to the scientific community; therefore, the *E. carinicauda* strain could be regarded as inbred when it has been mated brother×sister for twenty or more consecutive generations according to the criteria of mammal and fish inbred strains. The way of full-sibling mating has been handed down to the *F*_11_ generation of EC4 inbred line of *E. carinicauda.*

**Figure 1:**
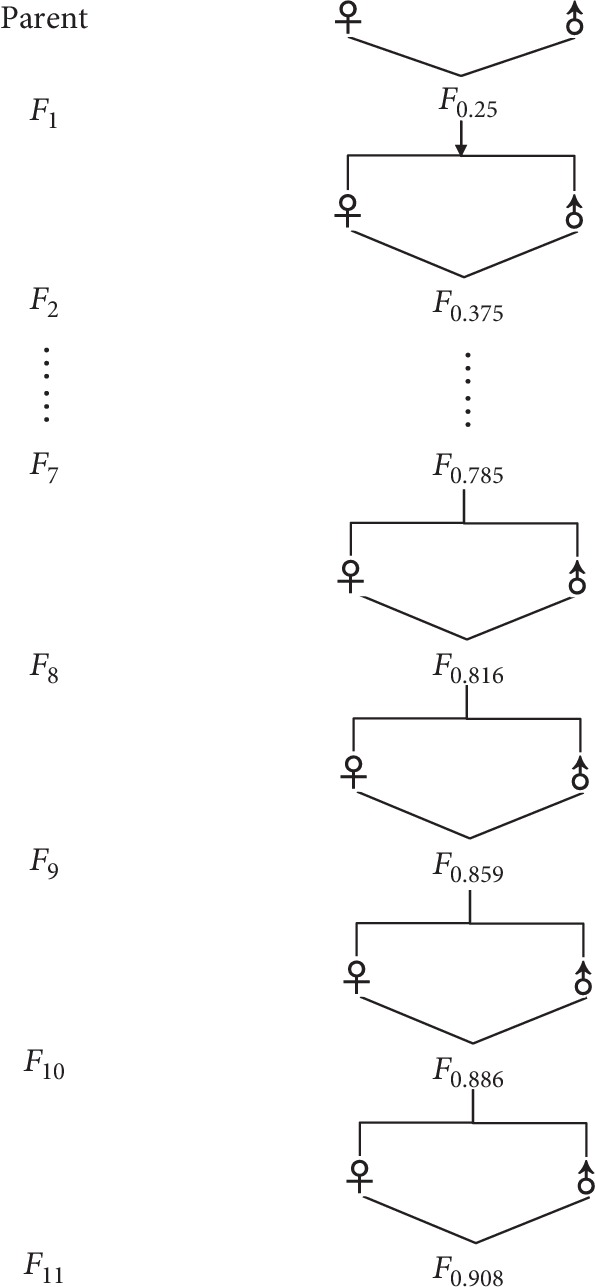
The inbred line establishment process for *E. carinicauda.*

The full-sibling family establishment process for EC4 inbred line is depicted in Figure 1 and by repeating the process, 11 generations of full-sibling family were obtained simultaneously until the 2017. As our previous study has shown, the *F*_1_ generation full-sibling family was established by brother×sister mating, and the *F*_2_ generation full-sibling family was obtained from the *F*_1_ generation with brother×sister mating, and the *F*_3_, *F*_4_, *F*_5_, *F*_6_, *F*_7_, *F*_8_, *F*_9_, *F*_10_, and *F*_11_ generations were obtained in a similar way [28]. Five groups (*F*_7_, *F*_8_, *F*_9_, *F*_10_, and *F*_11_) with different levels of inbreeding coefficient (*F* = 0.785, *F* = 0.816, *F* = 0.859, *F* = 0.886, and *F* = 0.908) were analyzed in this study. Every generation comes from a unique parent, and the population size of every generation was from 500 to 850. The inbreeding coefficient of each individual (*F*_*X*_) in a population can be calculated from Wright's equation devised by Sewell Wright [29]:

(1) $$\begin{array}{c} F_{X}=\sum \left\{{\left(\frac{1}{2}\right)}^{\left(n_{1}+n_{2}+1\right)}\left(1+F_{A}\right)\right\} \end{array}$$

where *n*_1_ is the intervening generations between the sire and the common ancestor, *n*_2_ is the intervening generations between the dam and the common ancestor, and *F*_*A*_ is the inbreeding coefficient of the common ancestor.

In the ninth generation of inbreeding population stage, a control population was established from the base breeding population, and all had an average inbreeding coefficient (*F*) of below 1%, which from the ten families (different males and females) hatched on the same day in order to increase the precision of the estimation, and the inbreeding coefficient of the control population was approximately 0.00.

The all postlarvae shrimps from every generation were transferred into 150 l polyvinyl chloride polymer (PVC) tanks containing 120 l aerated sand-filtered seawater and twenty shrimps in one tank with a temperature 25.0-26.0°C and initial salinity 31 ± 0.5 for rearing, respectively.

### 2.2. Experimental Design and Trait Measurement

A standardized procedure for family production was used in larvae rearing. Each inbred full-sibling and control group of fertilized eggs was hatched in separate tanks. The hatched larvae passed through six zoea stages to post larvae in 2 weeks at 25.0-26.0°C. A random sample of almost 100 post larvae from each generation was transferred into separate 5 larger tanks for rearing for reducing errors. We assessed the growth of the animals by recording the body weight and body length of all individuals alive, when the animals had reached ages of 60, 80, 100, and 120 days. The survival rate was also assessed during each of the four grow-out stages by dividing the number of shrimps stocked in each concrete pond at the beginning and the number found alive at the four grow-out stages.

The inbreeding depression coefficient (IDC) for all growth-related traits was evaluated as follows:

(2) $$\begin{array}{c} \text{IDC}=\frac{\left(1-\left(G_{\text{inbred}}/G_{\text{control}}\right)\right)}{\left(F_{\text{control}}-F_{\text{inbred}}\right)}\times 100\%, \end{array}$$

where *G*_control_ and *G*_inbred_ represent the mean body weight of the control shrimps and the inbreeding shrimps, and *F*_control_ and *F*_inbred_ represent the inbreeding coefficient of control population and the inbreeding population, respectively.

### 2.3. Genomic DNA Extraction

The thirty individuals of *E. carinicauda* were randomly sampled in the control population. The number of samples was from 36 to 51 according to the survival rate of the EC4 inbred line. Genomic DNA was extracted using the SDS method. Integrity of DNA was determined by 1% agarose gel electrophoresis and quantified by Nanodrop ND-1000 (Thermo Scientific, UK). The DNA was diluted to a working concentration of 50 ng/*μ*l.

### 2.4. Genotyping for SNP Markers

Picard-tools 1.41 and samtools 0.1.18 were used to sort, remove duplicated reads, and merge the bam alignment results of each sample. GATK2 software was used to perform SNP calling. Raw files were filtered with GATK standard filter method and other parameters, and only SNPs with distance > 5 were retained. Nucleotide sequences of fragments representing different SNP genotypes of ten shrimp were multiple-aligned using DNAMAN to determine whether shrimp carrying different SNP genotypes. Amplification refractory mutation system-quantitative PCR (ARMS-qPCR) was used as a tool to detect SNP loci.

### 2.5. EST-SSR Polymorphism Examination Analysis

There were 46 EST-SSR markers used to analyze the genetic structure and genetic diversity of the inbred and control populations. The sequences of the eight primers and microsatellite core sequences are shown in Table 1, and the other 38 EST-SSR markers are from Wang et al.'s study [30]. Primers were designed to flank the SSRs using Primer Premier 5.0 software (Premier Biosoft International, Palo Alto, CA, USA) and then synthesized by Shanghai Sangon Biological Engineering Technology (Shanghai, China). Polymerase chain reactions (PCRs) were performed in 20 *μ*l reaction volumes containing 50 ng of template DNA, 1 *μ*l of 10 *μ*M each primer, 10 *μ*l of 2× Reaction Mix, 0.2 *μ*l Golden DNA Polymerase (2.5 U/*μ*l), and 6.8 *μ*l sterile distilled water. The following PCR program included an initial step at 95°C for 4 min followed by 35 cycles of 95°C for 40 s, 55°C-60°C (depending on the Tm of the primer set used) for 40 s, 72°C for 1 min, and a final extension for 10 min at 72°C. Finally, the amplified products were detected by 3730XL sequencing test platform.

### 2.6. Sample Collections for the Immune Responses and Antioxidant Status Analysis

The postlarvae shrimps were transferred into 150 l polyvinyl chloride polymer (PVC) tanks containing 120 l aerated sand-filtered seawater with a temperature 25.0-26.0°C and initial salinity 31 ± 0.5 for rearing with the same feeding. Shrimps in the intermolt phase were sampled from the control population and each generation at 120 days, respectively. Ten shrimps (including 5 males and 5 females) were collected to sample haemolymph of each generation at 120-day growth stage. Meanwhile, haemolymph of ten shrimps was collected into a 2 ml sterile syringe containing an equal volume of anticoagulant solution (30 mM trisodium citrate, 0.34 M sodium chloride, and 10 mM EDTA-Na_2_, pH = 7.55) and then gently mixed in a sterile tube. A small part was immediately used to count the haemocytes and analyze phagocytic activity of the haemocytes, and the remainder was centrifuged at 1000× g for 10 min at 4°C, and the supernatant was dispensed into 2 ml Eppendorf tubes as cell-free haemolymph samples and stored at -80°C for analysis of other immune and antioxidant parameters. All assays for analyzing immune parameters were conducted in triplicate.

### 2.7. Determination of Immune Parameters

There were six immune parameters (the total haemocyte counts, the antibacterial activity, the haemocyanin (HEM) concentration, phenoloxidase (PO), lysozyme (LZM), and alkaline phosphatase (AKP) activity) and two antioxidant parameters (superoxide dismutase (SOD) and catalase (CAT) activity) were measured for analyzing the effective of inbreeding in different generations of EC4 inbred line according to the previous study [20, 31].

The total haemocyte counts were counted using Neubauer haemocytometer under a light microscope. A total of 200 *μ*l anticoagulant haemolymph was placed on the haemocytometer and the haemocytes were counted and expressed as cells ml^−1^ haemolymph.

The antibacterial activities of the haemolymph were measured according to the method of Ge et al. [31]. A total of 300 *μ*l bacterial suspension and 10 *μ*l cell-free haemolymph sample were pipetted into 96-well ELISA plate and the plate was put into microplate reader and shaken for a little while, and then OD570 nm was read and recorded as *A*_0_. Then, the plate was incubated in the microplate reader in dark at 37°C for 30 min and OD570 nm was recorded (*A*). The antibacterial activity was defined as *U*_*a*_ and then calculated as follows: *U*_*a*_ = (*A*_0_ − *A*)/*A*.

The absorbance of 100 *μ*l haemolymph mixed with 900 *μ*l sterile water was measured at 335 nm using Multiskan spectrum (Thermo, USA) to determine the haemocyanin (HEM) concentration and that was calculated using an extinction coefficient of 17.26.

Phenoloxidase (PO) activity of the haemolymph was measured spectrophotometrically at 490 nm by recording the formation of dopachrome produced from L-3,4-dihydroxyphenylalanine (L-DOPA, Sigma), according to the procedure described by Hernández-López et al. [32]. One unit of PO activity was defined as an increase in absorbance of 0.001 min^−1^ ml^−1^ cell-free haemolymph.

The activities of lysozyme (LZM), alkaline phosphatase (AKP), superoxide dismutase (SOD), and catalase (CAT) in cell-free haemolymph were measured using commercial kits (Jiancheng Bioengineering Institute, Nanjing, China) according to manufacturer's protocols. All assays for analyzing the above immune parameters were conducted in triplicate.

### 2.8. Statistical Analysis

Significant differences between the different generations were determined using one-way ANOVA and Tukey's multiple comparison test. Differences were considered significant at *P* < 0.05. Statistical computations were performed with IBM SPSS Statistics v22.

## 3. Result

### 3.1. The Distribution of the Identified SNPs from Transcriptome Sequences

A total of 827,145 SNPs were identified from the transcriptome sequencing data, containing 258,896 (31.30%) coding SNPs and 568,249 (68.70%) noncoding SNPs. The synonymous SNPs (201,079, 24.31%) were significantly more than that of no-synonymous SNPs (57,817, 6.99%). To verify the potential SNPs, a subset of 35 transcripts containing 151 putative SNPs were selected randomly for validation. Of these 151 primer pairs, 82 primers could produce reliable amplification, and the other 79 failed to amplify. The 31 SNP loci were polymorphic and considered as validated in the thirty individuals of the control population. The observed and expected heterozygosity ranged from 0.208 to 0.511 and from 0.186 to 0.833, with an average of 0.467 and 0.625, respectively. The minor allele frequency (MAF) ranged from 0.116 to 0.500, with an average of 0.382. The polymorphism information content (PIC) ranged from 0.184 to 0.375, with an average of 0.352 (Table 2).

### 3.2. The Genetic Characteristics of *F*_7_-*F*_11_ Detected with SNP and EST-SSR Loci

A total of 31 SNP markers and 46 EST-SSR markers were used to detect the dynamic change of genetic characteristics during the *E. carinicauda* inbreeding process from *F*_7_ to *F*_11_ generations in EC4 inbred line. The results showed that the number of alleles (*N*) gradually decreased with the increase of inbreeding generations, same as observed heterozygosity (*Ho*), expected heterozygosity (*He*), and PIC in Table 3 and Table 4. The *N*, *Ho*, *He*, and PIC from *F*_7_ to *F*_11_ generations of the EC4 inbred line were less than the control population which detected with SNP markers and EST-SSR markers.

The results showed that 9 of the SNP loci (29.03%, 9/31) became genetically homozygous in *F*_11_ generation of the EC4 inbred line. The 4 SNP loci including EcSNP017, EcSNP056, EcSNP092, and EcSNP098 loci were homozygous from *F*_7_ to *F*_11_ generations in the EC4 inbred line. The EcSNP072 locus was homozygous from *F*_8_ to *F*_11_ generations in the EC4 inbred line. The EcSNP037 and EcMLC-2 loci were heterozygous with 2 different alleles at the *F*_7_ and *F*_8_ generation, and then these loci became homozygous at the *F*_9_, *F*_10_, and *F*_11_ generations.

The results showed that 15 of the EST-SSR loci (32.61%, 15/46) became genetically homozygous in the *F*_11_ generation of the EC4 inbred line. The 6 EST-SSR loci including EC025, EC029, EC051, EC090, EC096, and EC160 were homozygous from *F*_7_ to *F*_11_ generations in the EC4 inbred line. The EC045 and EC080 were heterozygous in the *F*_7_ and *F*_8_ generations, and these loci became homozygous in the *F*_9_, *F*_10_ and *F*_11_ generations of EC4 inbred line. The four loci including EC059, EC052, EC182, and EC193 were heterozygous from *F*_7_ to *F*_9_ generations, and these loci became homozygous in the *F*_10_ and *F*_11_ generations of the EC4 inbred line.

The results showed that 5, 8, 10, 8, and 9 SNP loci were homozygous in *F*_7_, *F*_8_, *F*_9_, *F*_10_, and *F*_11_ generations, respectively; meanwhile, the 9, 8, 12, 13, and 15 EST-SSR loci were homozygous in *F*_7_, *F*_8_, *F*_9_, *F*_10_, and *F*_11_ generations, respectively. These results showed that the number of homozygous loci increased as the number of the generations increasing.

### 3.3. Effects of Inbreeding on the Growth-Related Traits of *E. carinicauda*

Effects of inbreeding on the body weight traits of *E. carinicauda* are shown in Table 5. The body weight of the control population was significantly higher than the EC4 inbred line of all generations at four growth stages (*P* < 0.05), except for *F*_7_ generation in EC4 inbred line at 120-day stage. In EC4 inbred line, the body weight of *F*_7_ generation is significantly higher than *F*_10_ and *F*_11_ generations at four growth stages (*P* < 0.05), and no statistically significant differences in body weight among *F*_8_, *F*_9_, *F*_10_, and *F*_11_ generations were found at all growth stages except for 100-day stage.

Effects of inbreeding on the body length traits of *E. carinicauda* are shown in Table 5. The body length of the control population was significantly higher than the EC4 inbred line of *F*_9_, *F*_10_, and *F*_11_ generations at four growth stages (*P* < 0.05). The body length of *F*_7_ generation was significantly higher than *F*_10_ and *F*_11_ generations at 60-, 80-, and 100-day stages (*P* < 0.05), and no statistically significant differences in body length among *F*_9_, *F*_10_, and *F*_11_ generations were found at 60-, 100-, and 120-day growth stages.

Effects of inbreeding on the survival rate traits of *E. carinicauda* are shown in Table 5. The survival rate of the control population was higher than all generations in EC4 inbred line. However, no statistically significant differences were found between the control population and *F*_7_ and *F*_8_ in the EC4 inbred line at all growth stages. The survival rate of the control population was significantly higher than the *F*_10_ and *F*_11_ generations in the EC4 inbred line at 100- and 120-day stage (*P* < 0.05). There were no statistically significant differences among the five generations of EC4 inbred lines at all growth stages.

As showed in Table 6, the estimated average inbreeding depression coefficient of body weight ranged from -18.17% to -26.75%, and the body length ranged from -4.19% to -9.72%, and the survival rate ranged from -6.02% to -26.03% per 10% increase of inbreeding coefficient of *F* at 120-day stage.

### 3.4. Effect of Inbreeding on the Immune Responses and Antioxidant Status of *E. carinicauda*

Effects of inbreeding on the immune parameters and antioxidant status of *E. carinicauda* are shown in Table 7. Inbreeding had no remarkable effect on the total haemocyte count, HEM concentration, and LZM activity. The antibacterial activity in the *F*_11_ generation was significantly lower than the *F*_7_ to *F*_10_ generations and control population (*P* < 0.05), and there were no significant differences between the control population and the *F*_7_ to *F*_10_ generations. The AKP activity of the *F*_10_ generation was significantly lower than the *F*_7_, *F*_8_, *F*_9_, and *F*_11_ generations and control population (*P* < 0.05), and there were no significant differences between the *F*_7_, *F*_8_, *F*_9_, and *F*_11_ generations and control population. The PO activity of the *F*_10_ and *F*_11_ generations was significantly lower than the *F*_7_, *F*_8_, and *F*_9_ generations and control population (*P* < 0.05), and there were no significant differences between the *F*_7_, *F*_8_, and *F*_9_ generations and control population.

The SOD activity in the *F*_11_ generation was significantly lower than *F*_7_ to *F*_10_ generations and control population (*P* < 0.05), and there were no significant differences of the SOD activity among the *F*_7_ to *F*_10_ generations and control population. The CAT activity of the *F*_10_ generation was significantly lower than the *F*_7_, *F*_8_, *F*_9_, and *F*_11_ generations and control population (*P* < 0.05), and there were no significant differences of the CAT activity among the *F*_7_, *F*_8_, *F*_9_, and *F*_11_ generations and control population.

## 4. Discussion

A strain shall be regarded as inbred when it has been mated brother×sister or offspring×parent mating for twenty or more consecutive generations at which point at least 98.6% of the loci in an individual of the strain will be homozygous [33]. However, full-sib mating is highly difficult to maintain due to inbreeding depression. Exceptionally, other breeding systems may be used, provided that the inbreeding coefficient achieved is at least equal to that at *F*_20_ [34]. In this study, the number of inbred lines was reduced from 8 to 2 until generation 11 using brother×sister mating, and the phenomenon of decrease in number of families during inbreeding of inbred line was also reported in other organisms. The number of families was reduced to 3 families until generation 5 in Japanese quail [35], and two strains were disappeared by generation 9 in *Xiphophorus helleri* [36], and there were 146 inbred strains cultivated but only 18 remained after twenty years in Yorkshire and Wessex [37]. Crnokrak and Roff have confirmed that inbred population frequently exhibits moderate to high levels of inbreeding depression in fitness traits [38]. Although the fertility, hatchability, and viability traits were not mentioned in these studies, the decreasing number of families was due to inbreeding depression for these characters.

Inbreeding depression, the decline in the value of a trait as a direct consequence of inbreeding, occurs in animal and plant populations. There was clear and irrefutable evidence for inbreeding depression in population [38, 39]. The genetic studies and molecular evolutionary approaches suggested that inbreeding depression was predominantly caused by the presence of recessive deleterious mutations in populations [40]. McCune et al. [41] found that there was at least 3.6 lethal equivalents of deleterious recessive alleles per zygote in inbred zebrafish, which results that the mortality was 9% in outbred zebrafish and 42% in inbred zebrafish between 6 and 48 days of age [41]. The small isolated populations are expected to accumulate inbreeding depression [42]. Maeda and Hashiguchi believed that the magnitude of inbreeding depression varies depended on the rapid loss of heterozygosity by successive brother-sister mating in inbred line population [35]. Furthermore, Frankham et al. (2001) found that deleterious alleles may be removed (purged) by natural selection in populations undergoing inbreeding. We have established the inbred line and also want to prevent depression of the beneficial trait. However, for all traits tested, the EC4 inbred line was decline compared with the control population except for some immune parameters in cell-free haemolymph. Inbreeding depression for different components of fitness was occurred in animals and plants, such as fertility [43], hatchability [44], body weight [45], and survival [46]. Studies about the inbreeding depression of aquaculture species are of general concern, including *Litopenaeus vannamei* [19, 23, 47], *Eulimnadia Texana* [48], *Penaeus (marsupenaeus) japonicas* [25], *Fenneropenaeus chinensis* [24], and *Portunus trituberculatus* [20, 49, 50].

The effects of inbreeding depression have been repeatedly identified in diverse aquaculture species, but the impact and direction of these effects have been inconsistent. This study found that inbreeding had a significant negative effect on body weight and body length at harvest size (120 days of age) with an estimated effect on inbreeding coefficient. This is consistent to the estimated by Ríos-Pérez et al. who found that inbreeding had a significant negative effect on the body weight at the age of 130-day stage in *Penaeus (Litopenaeus) vannamei* [19]. Significant inbreeding depression was found at all the inbreeding levels studied (80, 100, and 140 days), the inbreeding depression in the body weight at the 140-day stage was found to be -10.4% at *F* = 0.25, -16.61% at *F* = 0.375, and -23.68% at *F* = 0.50, and an increasing inbreeding depression of growth was observed with increasing inbreeding coefficient at 140-day stage in *Fenneropenaeus chinensis* [24].

However, our results differed from some reports. Keys et al. found an estimated effect of -3.34% of growth trait per 10% increase on inbreeding coefficient, but not statistically significant in *Penaeus japonicus* [25]. Moss et al. found that inbreeding had no effect on grow-out trait in *Penaeus (Litopenaeus) vannamei* [23]. In a retrospective observational study, Moss et al. estimated that inbreeding had a small but significant effect on growth trait that ranged from -2.6 to -3.9% of the phenotypic mean per 10% increase on inbreeding coefficient, which is different from his previous study but similar to our findings [47]. Inbreeding is considered to affect fitness traits which also including survival rate [40]; our results showed that inbreeding had a significant effect on survival rate in EC4 inbred line. Similarly, Moss et al. found that inbreeding had a significant effect on grow-out survival in *P. vannamei* [23], and Luo et al. estimated that the average inbreeding depression was found to be -5.95% for survival at 80-day stage, -5.51% at 100-day stage, and -6.71% at 140-day stage [24]. In contrast, Keys et al. estimated inbreeding depression coefficients of -3.43% per 10% increase for survival at three different growth stages, even though not statistically significant [25], and Moss et al. also found that inbreeding had no effect on grow-out survival rate in *P. vannamei* [47]. The use of a relatively low number of families in these studies brought about the contradictory results. We also believed that it is difficult to keep the environment constant in the different generation. Therefore, more repetitions, more different inbreeding population, and more generations were needed to increase the accuracy and precision of this study.

The crustaceans lack an adaptive immune system and rely entirely on an efficient innate immune system to defend themselves from pathogen invasions [51, 52]. The antibacterial activity can reflect humoral responses due to bacterial killing, and the shrimp could resist the foreign pathogens by improving the antibacterial activity [53]. Numerous studies have found that PO plays a critical function in immune defense of invertebrate [54]. Fagutao et al. found that PO not only regulates other immune-related genes and defends against pathogenic microorganisms but also maintains normal functioning and is thus essential for survival in *Marsupenaeus japonicus* [55]. In this study, the antibacterial activity and PO activity in the *F*_11_ generation were significantly lower than that in the control population of *E. carinicauda* (*P* < 0.05), and the AKP activity in the *F*_10_ generation was significantly lower than that in the control population. Ren et al. also found that inbreeding could affect the antibacterial activity in the haemolymph of crabs [20].

The aquatic animals are susceptible to oxidative stress, which has an efficient antioxidant defense system to prevent oxidative stress and maintain a balanced cellular redox state [56]. The antioxidant defense system consists of a cascade of enzymes (including SOD and CAT) and nonenzymatic small antioxidant molecules [57, 58]. Mai et al. found that SOD has important antibacterial and antiviral function in *M. japonicus* [59]. In this study, the SOD activity in the *F*_11_ generation was significantly lower than that in the control population, and the CAT activity in the *F*_10_ generation was significantly lower than that in the control population (*P* < 0.05). Overall, our data suggests that the immune and antioxidant defense systems of the *F*_10_ or *F*_11_ were less effective compared to the control population except the total haemocyte count, HEM concentration, and LZM activity.

It has been thought that *E. carinicauda* would be difficult for us to establish an inbred line because of its more genetic variation, 90 chromosomes [60], and possesses a large complex genome (5.73 Gb) [10]. Therefore, it is necessary to detect the genetic characteristics of inbred line from a relatively low generation to the current generation by SNP and EST-SSR markers [61, 62]. In order to obtain accurate estimates within 0.05 of the population allele frequency with high probability (≥95%), a sample size of >30 is often required [63]. In the present study, the number of samples was from 36 to 51 according to the survival rate of the population. The EST-SSR markers used in this study were polymorphic, and the PIC in the control population was 0.667, and 35 (76.09%) markers were high polymorphic marker. The 46 polymorphic EST-SSR loci were used to analyze to detect 296 alleles in control population, 96 alleles in the *F*_11_ generation of EC4 inbred lines, indicating that a large number of alleles had been lost during inbreeding. Du et al. found that 26 out of 28 microsatellite loci were homozygous in *F*_20_ of *Mongolian gerbils* [61]. The average number of alleles, especially when compared to control population, indicates that there were lost in the genetic variability in the raise of these inbred lines.

The inbreeding coefficient of an individual is the probability that both alleles at a locus are identical by descent [64], which was usually used to measure inbreeding. As the *F* is a probability, it ranges from 0 to 1, the former being outbred and the latter completely inbred. The EC4 inbred line was established using by brother×sister mating, and the *F* was 0.908 for generation 11 which closing to 1. However, our results could lower than the theoretical estimate which was inferred by the genetic data. It is probably that there was wide variation in homozygosity among loci due to the chance effects involved in Mendelian segregation. Consequently, the more polymorphic loci should be used to obtain a reliable estimate of the effective inbreeding coefficient for an individual. Another reason was probably that deleterious alleles may be purged by artificial selection.

In summary, the number of alleles (*N*), observed heterozygosity (*Ho*), expected heterozygosity (*He*), and polymorphism information content (PIC) gradually decreased with the increase of inbreeding generations. Inbreeding had a small but significant effects on body weight and body length in the *F*_9_, *F*_10_, and *F*_11_ generations and had a significant effect on survival rate of *F*_11_ generation of EC4 inbred line. There was a significant generation dependence in the immune parameters of antibacterial activity, AKP and PO activities, and antioxidant indexes of SOD and CAT activities.

Although we spent almost 10 years to establish this new inbred strain, the number of inbred lines was reduced from 8 to 2 by generation 11 using brother×sister mating due to inbreeding depression of fertility, hatchability, and viability. We will do some more to research the nutritional requirement of inbred line and the behavior characteristics, biochemical indices, and physiological indices. We will investigate these questions in the future and reveal and enrich more knowledge about EC4 inbred line of *E. carinicauda*.

## Figures and Tables

**Table 1:** Eight polymorphic EST-SSR markers and estimated polymorphisms.

Loci		Repeat motif	*Na*	*Ho*	*He*	PIC	GenBank ID
EC002	GCCATAACAAGTTCCAACCTAA	(GA)_12_	11	0.933	0.871	0.843	MG976898
	ACCAGACTGCTCTCCCACATA						

EC034	ACTTCATCCACAAGCAGAGGT	(ATC)_7_	7	0.517	0.724	0.720	KX394706
	GAAGAAGAGGAAGGTGGGGC						

EC096	GCAATTTGCCTGTTCGGTCT	(TTG)_6_	6	0.367	0.597	0.664	KX394741
	GGTAGGGGTAAGGGGGTGAT						

EC205	CTACCAAAGAGCAAATGGAGTC	(CT)_30_	10	0.967	0.871	0.840	MG976900
	TGAAGTGCTTGGAGTAAAAGATT						

EC208	GGACGCTTGTTACAACCTCGAT	(CTT)_12_	8	0.667	0.840	0.802	MG976894
	TTCAGCCTTAGCCATCAGTAAGA						

EC212	CGTAGTAGTAATAAAGCGACCCA	(AC)_14_	7	0.586	0.759	0.709	MG976897
	TCGGGCTTGTCTTACTTACCT						

EC216	ATCATGAGGAGGACATTGCGT	(GGA)_14_	7	0.575	0.798	0.758	MG976893
	GACTTGACCCTGTTCCTGCC						

EC219	GTTTGTCCAAACTCCGAGTGAAA	(CTT)_23_	8	0.767	0.845	0.804	MG976895
	CATCGCAACACCGACAGACA						

**Table 2:** Characteristics of the 31 SNP markers for *E. carinicauda.*

SNP loci	SNP type	*Ho*	*He*	PIC	MAF	Annotation
EcSNP011	A/C	0.484	0.651	0.364	0.395	MAP kinase-interacting serine/threonine-protein
EcSNP017	C/T	0.493	0.558	0.368	0.419	Actin cytoskeleton organization and biogenesis protein
EcSNP020	A/C	0.507	0.833	0.368	0.417	Rhophilin Rho GTPase-binding protein 1
EcSNP037	A/G	0.436	0.442	0.338	0.314	Ras-like GTP-binding protein Rho1
EcSNP056	C/T	0.488	0.767	0.366	0.407	Inositol hexakisphosphate kinase
EcSNP057	G/T	0.444	0.512	0.343	0.326	Inositol hexakisphosphate kinase
EcSNP063	A/G	0.488	0.674	0.366	0.407	Unknown
EcSNP072	A/G	0.208	0.186	0.184	0.116	Heat shock protein 70
EcSNP092	A/C	0.361	0.326	0.293	0.233	Skeletal receptor tyrosine protein kinase
EcSNP098	C/T	0.460	0.419	0.351	0.349	Skeletal receptor tyrosine protein kinase
EcSNP105	A/G	0.503	0.791	0.374	0.465	Unknown
EcSNP131	C/T	0.510	0.792	0.375	0.479	Unknown
EcSNP137	C/T	0.391	0.500	0.305	0.250	Condensin complex subunit 2
EcSNP155	A/G	0.427	0.372	0.333	0.302	Heat shock protein 70
EcSNP161	A/G	0.506	0.674	0.375	0.500	Heat shock protein 70
EcSNP177	C/T	0.488	0.674	0.366	0.407	Unknown
EcSNP182	C/T	0.502	0.581	0.373	0.454	Heat shock protein 70
EcCat-2	A/G	0.497	0.833	0.368	0.417	Cathepsin L
EcFABP-2	C/T	0.507	0.833	0.373	0.458	Fatty acid-binding protein
EcMHC-4	C/T	0.489	0.625	0.364	0.396	Myosin heavy chain type 2
EcMHC-5	A/G	0.497	0.833	0.368	0.417	Myosin heavy chain type 2
EcMHC-6	C/G	0.507	0.583	0.373	0.458	Myosin heavy chain type 2
EcMHC-7	A/G	0.503	0.458	0.371	0.438	Myosin heavy chain type 2
EcMLC-2	C/T	0.479	0.750	0.359	0.375	Myosin light chain
EcMLC-4	C/T	0.497	0.583	0.368	0.417	Myosin light chain
EcTroC-4	C/T	0.403	0.542	0.317	0.271	Troponin C
EcSmad3-3	A/G	0.511	0.833	0.375	0.500	Smad3
EcTGFI-6	G/T	0.467	0.625	0.353	0.354	TGF-beta-induced protein
EcTGFI-10	C/T	0.479	0.667	0.359	0.375	TGF-beta-induced protein
EcTGFI-15	C/T	0.489	0.792	0.364	0.396	TGF-beta-induced protein
EcBMP2-3	C/T	0.454	0.667	0.346	0.333	Bone morphogenetic protein 2
Max		0.511	0.833	0.375	0.500	
Min		0.208	0.186	0.184	0.116	
Mean		0.467	0.625	0.352	0.382	

Note: *Ho*: observed heterozygosity; *He*: expected heterozygosity; PIC: polymorphism information content; MAF: minor allele frequency.

**Table 3:** Genetic characteristics of control population and EC4 inbred line with SNP markers in *E. carinicauda.*

Group	*N*	*Ho*	*He*	PIC
Control population	2.000^a^	0.625^a^	0.467^a^	0.352^a^
*F*_7_	1.839^ab^	0.318^b^	0.288^b^	0.218^b^
*F*_8_	1.742^b^	0.290^b^	0.242^b^	0.191^b^
*F*_9_	1.677^b^	0.293^b^	0.243^b^	0.189^b^
*F*_10_	1.742^b^	0.301^b^	0.255^b^	0.199^b^
*F*_11_	1.710^b^	0.253^b^	0.211^b^	0.175^b^

Note: *N*: the number of alleles.

**Table 4:** Genetic characteristics of control population and EC4 inbred line with EST-SSR markers in *E. carinicauda.*

Group	*N*	*Ho*	*He*	PIC
Control population	6.435^a^	0.545^a^	0.652^a^	0.667^a^
*F*_7_	2.239^b^	0.252^b^	0.304^b^	0.254^b^
*F*_8_	2.130^b^	0.250^b^	0.269^b^	0.232^bc^
*F*_9_	2.217^b^	0.252^b^	0.275^b^	0.204^bc^
*F*_10_	2.174^b^	0.243^b^	0.253^b^	0.180^c^
*F*_11_	2.087^b^	0.229^b^	0.236^b^	0.174^c^

**Table 5:** Comparative analysis of growth-related traits between EC4 inbred line and control population.

Trait	Group	Growth stages			
		60 days	80 days	100 days	120 days
Body weight (g)	Control population	0.60 ± 0.27^a^	1.03 ± 0.32^a^	1.44 ± 0.51^a^	1.73 ± 0.57^a^
	*F*_7_	0.53 ± 0.23^b^	0.89 ± 0.26^b^	1.22 ± 0.41^b^	1.51 ± 0.43^ab^
	*F*_8_	0.44 ± 0.16^bc^	0.85 ± 0.30^bc^	1.20 ± 0.38^b^	1.46 ± 0.41^bc^
	*F*_9_	0.46 ± 0.15^bc^	0.74 ± 0.18^c^	1.12 ± 0.31^b^	1.46 ± 0.21^bc^
	*F*_10_	0.40 ± 0.11^c^	0.72 ± 0.18^c^	0.93 ± 0.21^c^	1.32 ± 0.25^c^
	*F*_11_	0.41 ± 0.13^c^	0.70 ± 0.22^c^	0.96 ± 0.17^c^	1.36 ± 0.22^c^

Body length (mm)	Control population	33.70 ± 4.58^a^	40.69 ± 4.43^a^	45.05 ± 5.84^a^	48.74 ± 5.83^a^
	*F*_7_	32.87 ± 4.72^ab^	39.47 ± 3.31^ab^	43.57 ± 4.87^ab^	47.19 ± 4.98^ab^
	*F*_8_	30.65 ± 4.21^c^	38.46 ± 3.82^bc^	43.60 ± 4.80^ab^	46.01 ± 4.71^ab^
	*F*_9_	31.01 ± 3.79^bc^	36.49 ± 3.11^c^	41.40 ± 3.85^bc^	45.42 ± 2.25^b^
	*F*_10_	29.58 ± 2.62^c^	35.88 ± 3.08^c^	39.72 ± 3.23^c^	44.96 ± 2.92^b^
	*F*_11_	29.60 ± 2.72^c^	35.91 ± 3.15^d^	40.33 ± 2.48^c^	44.43 ± 2.63^b^

Survival rate (%)	Control population	95.00 ± 1.73^a^	82.00 ± 2.00^a^	68.00 ± 2.83^a^	55.00 ± 3.32^a^
	*F*_7_	90.00 ± 3.54^ab^	73.75 ± 2.17^ab^	62.50 ± 2.50^ab^	48.75 ± 2.17^ab^
	*F*_8_	88.75 ± 5.45^ab^	74.55 ± 9.07^ab^	66.25 ± 3.78^ab^	52.39 ± 6.86^ab^
	*F*_9_	86.67 ± 2.36^b^	72.22 ± 1.96^b^	59.72 ± 1.96^b^	47.22 ± 1.96^ab^
	*F*_10_	90.00 ± 2.00^ab^	73.00 ± 1.73^ab^	60.00 ± 4.00^b^	44.00 ± 2.83^b^
	*F*_11_	86.00 ± 3.74^b^	70.00 ± 3.16^b^	59.00 ± 3.16^b^	42.00 ± 3.74^b^

**Table 6:** Inbreeding depression on growth-related traits of five levels of inbreeding in EC4 inbred line at 120-day stage.

Group	IDC (body weight)	IDC (body length)	IDC (survival rate)
*F*_7_	-16.20%	-4.19%	-14.59%
*F*_8_	-19.13%	-6.79%	-6.02%
*F*_9_	-18.17%	-7.89%	-16.51%
*F*_10_	-26.75%	-8.81%	-22.57%
*F*_11_	-23.55%	-9.72%	-26.03%

Note: IDC: the inbreeding depression coefficient.

**Table 7:** The immune parameters and antioxidant status of control population and different inbreeding generations in *E. carinicauda.*

Generation	Total haemocyte count	Antibacterial activity	HEM concentration	AKP activity	LZM activity	PO activity	SOD activity	CAT activity
	(×10^7^/ml)	(U)	(mg/ml)	(U/ml)	(U/ml)	(U)	(U)	(U/ml)
Control population	5.86 ± 0.14^a^	0.48 ± 0.02^a^	6.36 ± 0.09^a^	6.66 ± 0.17^a^	0.40 ± 0.01^a^	0.40 ± 0.02^a^	6.92 ± 0.17^a^	16.40 ± 0.41^a^
*F*_7_	5.89 ± 0.12^a^	0.48 ± 0.03^a^	6.37 ± 0.16^a^	6.71 ± 0.18^a^	0.40 ± 0.03^a^	0.41 ± 0.02^a^	6.69 ± 0.23^a^	15.33 ± 0.58^a^
*F*_8_	5.72 ± 0.16^a^	0.47 ± 0.02^a^	6.49 ± 0.34^a^	6.64 ± 0.21^a^	0.37 ± 0.03^a^	0.40 ± 0.02^a^	6.95 ± 0.31^a^	15.89 ± 1.01^a^
*F*_9_	5.43 ± 0.37^a^	0.46 ± 0.02^a^	6.21 ± 0.23^a^	6.68 ± 0.20^a^	0.39 ± 0.01^a^	0.38 ± 0.01^a^	6.74 ± 0.26^a^	15.77 ± 0.52^a^
*F*_10_	5.52 ± 0.23^a^	0.47 ± 0.01^a^	5.87 ± 0.27^a^	6.15 ± 0.16^b^	0.39 ± 0.02^a^	0.34 ± 0.01^b^	6.52 ± 0.18^a^	12.96 ± 0.85^b^
*F*_11_	5.63 ± 0.20^a^	0.40 ± 0.01^b^	6.07 ± 0.17^a^	6.52 ± 0.25^a^	0.38 ± 0.02^a^	0.30 ± 0.01^c^	6.01 ± 0.25^b^	14.70 ± 0.91^a^

Note: HEM: haemocyanin; AKP: alkaline phosphatase; LZM: lysozyme; PO: phenoloxidase; SOD: superoxide dismutase; CAT: catalase.

## Data Availability

The data used to support the findings of this study are included within the article.
